# Pure red cell aplasia and anti-erythropoietin antibodies in patients on hemodialysis: a report of two cases and a literature review

**DOI:** 10.1590/2175-8239-JBN-2018-0054

**Published:** 2018-08-23

**Authors:** Gabriela Lacreta, Sérgio Gardano Elias Bucharles, Gabriela Sevignani, Miguel Carlos Riella, Marcelo Mazza do Nascimento

**Affiliations:** 1 Universidade Federal do Paraná, Hospital de Clínicas, Serviço de Nefrologia, Curitiba, PR, Brasil.; 2 Fundação Pró Renal, Curitiba, PR, Brasil.; 3 Clínica Evangélico de Hemodiálise, Curitiba, PR, Brasil.; 4 Universidade Federal do Paraná, Hospital de Clínicas, Curitiba, PR, Brasil.

**Keywords:** Anemia, Renal Insufficiency, Chronic, Renal Dialysis, Anemia, Insuficiência Renal Crônica, Diálise Renal

## Abstract

**Introduction:**

Anemia is a frequent multifactorial complication of CKD seen in patients on dialysis derived mainly from impaired erythropoietin (EPO) production. A less common cause of anemia in individuals with CKD is pure red cell aplasia (PRCA) secondary to the production of anti-EPO antibodies.

**Objective:**

This paper aimed two describe two cases of PRCA secondary to the production of anti-EPO antibodies including choice of treatment, patient progression, and a literature review.

**Materials:**

This study included the cases of two patients with CKD on hemodialysis with severe anemia in need of specific investigation and management.

**Results:**

Patient 1 with CKD secondary to hypertension treated with EPO for 7 months showed persistent decreases in hemoglobin (Hb) levels despite the subcutaneous administration of increasing doses of EPO; the patient required recurring blood transfusions. Workup and imaging tests were negative for the main causes of anemia in individuals with CKD on dialysis. Patient 2 with CKD secondary to adult polycystic kidney disease had been taking EPO for 2 years. The patient developed severe abrupt anemia the month he was started on HD, and required recurring transfusions to treat the symptoms of anemia. Workup and imaging findings were inconclusive. Specific laboratory tests confirmed the patients had anti-EPO antibodies. After six months of immunosuppressant therapy (corticosteroids + cyclosporine) the patients were stable with Hb > 9.0 g/dl.

**Conclusion:**

PRCA is a rare condition among patients on dialysis treated with rhEPO and should be considered as a possible cause of refractory anemia. Treating patients with PRCA may be challenging, since the specific management and diagnostic procedures needed in this condition are not always readily available.

## RESUMO

**Introdução:**

Anemia é complicação frequente da Doença Renal Crônica (DRC) em pacientes dialíticos. Apresenta caráter multifatorial principalmente pela insuficiente produção de eritropoietina (EPO). Situação rara causadora de anemia na DRC é Aplasia Pura de Células Vermelhas (APCV), em decorrência da produção de anticorpos anti-EPO.

**Objetivo:**

Descrever 2 casos de APCV com formação de anticorpos anti-EPO, sua abordagem clínica, evolução e revisão de literatura.

**Métodos:**

Dois pacientes em hemodiálise que desenvolveram anemia grave, necessitando investigação e manejo específico.

**Resultados:**

Paciente nº 1: feminina, 75 anos, DRC secundária à hipertensão arterial. Após 7 meses com EPO desenvolveu queda persistente em valores de hemoglobina (Hb) mesmo com incremento em doses EPO SC, necessitando transfusões de sangue recorrentes. Extensa investigação laboratorial e de imagem resultou negativa para principais causas de anemia. Paciente nº 2: masculino, 66 anos, DRC secundária à DRPA, há 2 anos em uso de EPO. No mês de entrada em HD desenvolveu anemia severa, também exigindo transfusões recorrentes para tratamento da anemia sintomática. Extensa investigação laboratorial e por imagem, sem chegar a uma conclusão definitiva. Em ambos os casos a presença de anticorpos anti-EPO foi confirmada por exames laboratoriais específicos. Terapia imunossupressora resultou em estabilização do quadro e Hb > 9,0 g/dl em ambos os pacientes, 6 meses após início do tratamento.

**Conclusão:**

APCV é condição rara entre pacientes dialíticos que recebem EPOHuR e deve ser lembrada como causa de anemia refratária. Seu manejo específico e diagnóstico laboratorial nem sempre acessível, tornando desafiadora a condução dos casos para o nefrologista.

## INTRODUCTION

Anemia is a frequently observed complication in patients with chronic kidney disease (CKD) and individuals on chronic dialysis in particular.^1^^,^ 2 Anemia is defined as the presence of hemoglobin levels below 12 g/dl and 13 g/dl in females and males, respectively. Prevalence grows throughout the various stages of CKD to reach values above 50% among patients with glomerular filtration rates < 15 ml/minute/1.73m^2^.^3^ The presence of anemia in CKD contributes to decreased quality of life, increased risk of hospitalization, and cognitive impairment, not to mention associations with severe complications such as cardiovascular disease and increased mortality.^4^

Although anemia in CKD is primarily caused by impaired production of erythropoietin (EPO), various other clinical conditions and concurrent diseases may contribute to the onset of anemia in individuals with advanced CKD, such as functional or absolute iron deficiency, chronic infection, systemic inflammation, inadequate dialysis, digestive blood loss, specific vitamin deficiency (vitamin B12 and folate), in addition to osteitis fibrosa cystica and hemoglobinopathy concomitant to lack of response to stimulation with EPO. 

EPO is normally produced in the interstitial fibroblasts of the renal cortex, tubular epithelial cells, and peritubular capillaries.^5^ In structural terms, EPO is a glycoprotein hormone composed by a chain of 165 amino acids and carbohydrates - an essential feature for the in vivo biological function of EPO, since partially or completely deglycosylated EPO is rapidly degraded in the body.^5^ EPO stimulates the production of red cells by binding to homodimer receptors located in primitive erythroid progenitors and colony forming units - erythroid, preventing the apoptosis of these cell types and of the initially formed erythroblasts and allowing cell division and the maturation of red blood cells.^5^

Anemia in CKD is typically normocytic and normochromic, without leukopenia or thrombocytopenia. Mean survival and the production of red cells are decreased in CKD settings, although the latter is more important.^2^ Serum EPO levels are in general normal or discretely elevated in patients with CKD and anemia, but are deemed exceedingly low in relation to the degree of anemia, since anemic patients with preserved renal function have EPO levels 10 to 100 times higher.^6^^,^ 7

One of the less frequent causes of anemia in individuals with CKD, pure red cell aplasia (PRCA) associated with EPO therapy was first described in the medical literature in the 1980s and 1990s. PRCA has attracted the interest of nephrologists on account of its challenging clinical characteristics and diagnostic and therapeutic peculiarities.^8^ PRCA is a rare hematologic condition characterized by sudden onset severe progressive normocytic normochromic anemia, low reticulocyte count (< 10.000/mm^3^), and nearly complete absence of erythroid precursor cells in the bone marrow.^9^ Patients on chronic dialysis and erythropoiesis-stimulating agents may have PRCA secondary to the formation of neutralizing anti-EPO antibodies, which block the interaction of EPO and its receptors^10^ by neutralizing all exogenous EPO and cross-reacting with endogenous EPO.

This paper aimed to describe two cases of PRCA induced by the use of recombinant human erythropoietin (rhEPO) infused subcutaneously in patients with chronic kidney disease on hemodialysis at a renal replacement therapy center in Curitiba, Brazil; the paper also aimed to perform a review on PRCA to help nephrologists better recognize this condition and shed light on possible therapeutic approaches.

## CLINICAL CASES

### CASE 1

A.G., a retired 75-year-old Caucasian female residing in Curitiba with a history of CKD secondary to hypertensive nephrosclerosis, had been managed conservatively for nearly two years. Two months prior to the start of renal replacement therapy (January 2015), she was started on an erythropoiesis-stimulating agent (epoetin alfa); at the time, her hemoglobin (Hb) level was below 10 g/dl.

At the start of hemodialysis (March 2015, Figure 1), the patient was anemic (Hb = 8.5 g/dl) and with absolute iron deficiency (serum ferritin: 10.1 ng/ml; serum iron: 20,6 µg/dl; transferrin saturation: 4.3%). She was started on intravenous iron hydroxide and the dose of subcutaneous EPO was escalated to a weekly 12,000 IU. Her workup improved, with hemoglobin reaching 12,0 g/dl in May 2015 and iron stores normalizing (serum iron: 156.1 µg/dl; ferritin: 409 ng/ml; and transferrin saturation: 45.3%), while dialysis adequacy was satisfactory (KT/V > 1.2). Between July and October 2015, while on EPO therapy, her hemoglobin dropped significantly and gradually to below 8,0 g/dl despite changes in EPO dosage, which prompted the need for blood transfusions on account of symptomatic anemia and hospitalization to further investigate the causes of anemia unresponsive to EPO.

**Figure 1:**
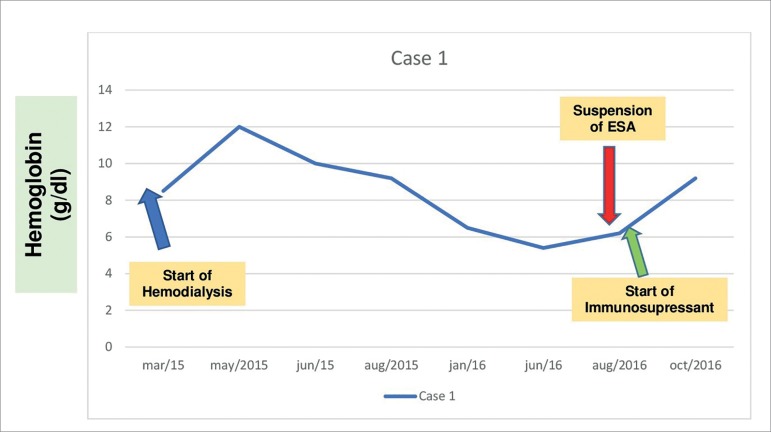
Timeline showing hemoglobin levels (g/dl) and main clinical events - Case 1.

On the second semester of 2015 and on the first semester of 2016 the patient underwent examination by upper gastrointestinal (UGI) endoscopy and colonoscopy, which revealed she was not suffering from GI bleeding. Direct and indirect Coombs tests were negative for hemolysis; her reticulocyte count was low (< 10,000 / µL), while bilirubin and lactate dehydrogenase levels were normal; her parathyroid hormone level was < 500 pg/ml (Table 1). Despite treatment with EPO in doses > 50 UI/kg 3x/week (12,000 IU a week), the patient had persistent asthenia and weakness associated with low hemoglobin levels and required monthly blood transfusions.

**Table 1:** Clinical investigation and laboratory tests at the time of diagnosis of PRCA - Case 1

Tests	Results	Reference Values
Hemoglobin	5.8 g/dl	12.5-15.7 g/dl
Hematocrit	18.2%	36-46%
Iron	219 µg/dl	5-170 µg/dl
Ferritin	1295.9 ng/ml	4.6-204 ng/ml
Transferrin saturation	59.4%	15-50%
Reticulocyte count	8,400 µL	22,500-147,000 / µL
Lactate dehydrogenase	130 U/L	125-220 U/L
Vitamin B12	1001 pg/ml	187-883 pg/ml
Folic acid	14.6 ng/ml	3.1-20.5 ng/ml
Total bilirubin	0.47 mg/dl	0.1-1.2 mg/dl
C-reactive protein	6.78 mg/dl	< 0.50 mg/dl
Serum albumin	3.2 g/dl	3.5-5.0g/dl
Parathyroid hormone	412 pg/ml	15-68 pg/ml
TSH	2.17 µUI/ml	0.35-4.94 µUI/ml
Glutamic-pyruvic transaminase	11 U/L	0-55 U/L

In June 2016, after extensive blood testing, bone marrow examination with myelography found erythroid hypoplasia and normal presentations in the other cell series (granulocytic, lymphocytic, and platelet) without infiltration of neoplastic cells. The analysis of all information available suggested the patient had PRCA associated with anti-EPO antibodies. In July 2016 she was tested for anti-EPO antibodies and EPO therapy was suspended. On the following month test results showed she was positive for neutralizing anti-EPO antibodies. Treatment with EPO was discontinued and the patient was started on immunosuppressant therapy (prednisone 1 mg/kg/day with further de-escalation; and cyclosporine 4 mg/kg/day, divided in two doses).

Hemoglobin levels improved gradually after the introduction of immunosuppressant therapy. Values reached their best levels between September and October of 2016 (9.2 g/dl), and since then the patient has not been offered packed red blood cells. Immunosuppressant therapy was discontinued after four months on account of side effects (esophageal candidiasis and persistent hand tremors). Nevertheless, hemoglobin levels have been stable and the patient has been off epoetin alfa.

### CASE 2

**Figure 2:**
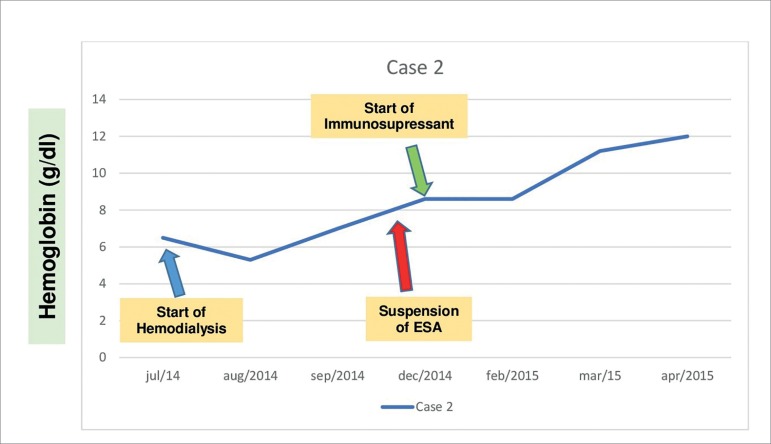
Timeline showing hemoglobin levels (g/dl) and main clinical events - Case 2.

J.W.S.A., a 66-year-old male Caucasian physician residing in Curitiba, Brazil, had advanced CKD secondary to adult polycystic kidney disease. He had been managed conservatively for five years and started hemodialysis (HD) in June 2014 (Figure 2). At the time he had been taking epoetin alfa regularly for two years, and in the month after the start of HD he was found to have severe sudden onset anemia (Hb < 7.0 g/dl) with normal iron stores (serum iron: 58.5 µg/dl; ferritin: 380 µg/L; and transferrin saturation: 20.9%). 

Endoscopic examination (UGI endoscopy and colonoscopy) did not reveal sources of active bleeding. Between July and December 2014, the patient needed monthly transfusions with packed red blood cells due to symptomatic anemia. He underwent the same diagnostic examination procedures described for the patient discussed in Case 1 (Table 2), including bone marrow testing, which showed erythroid hypoplasia and normal presentations in the other cell series (granulocytic, lymphocytic, and platelet). In October 2014, PRCA related to anti-EPO antibodies was considered. Blood tests revealed the patient was positive for neutralizing anti-EPO antibodies. The patient was taken off epoetin alfa in November 2014 and was started on cyclosporine and prednisone at doses similar to the ones described for the patient in Case 1. His hemoglobin levels began to improve gradually since January 2015. He no longer needed transfusions and was kept on cyclosporine until May 2015 as recommended by the hematology team, when he underwent kidney transplantation (deceased donor). The patient is currently well.

**Table 2:** Clinical investigation and laboratory tests at the time of diagnosis of PRCA - Case 2

Tests	Results	Reference Values
Hemoglobin	7.0 g/dl	12.5-15.7 g/dl
Hematocrit	20.1%	36-46%
Iron	226 µg/dl	5-170 µg/dl
Ferritin	1500 ng/ml	4.6-204 ng/ml
Transferrin saturation	105%	15-50%
Reticulocyte count	9.300 µL	22.500-147.000 / µL
Lactate dehydrogenase	311 U/L	125-220 U/L
Vitamin B12	308 pg/ml	187-883 pg/ml
Folic acid	13.0 ng/ml	3.1-20.5 ng/ml
Total bilirubin	0.71 mg/dl	0.1-1.2 mg/dl
C-reactive protein	5.45 mg/dl	< 0.50 mg/dl
Serum albumin	4.3 g/dl	3.5-5.0g/dl
Parathyroid hormone	148 pg/ml	15-68 pg/ml
TSH	3.7 µUI/ml	0.35-4.94 µUI/ml
Glutamic-pyruvic transaminase	20 U/L	0-55 U/L

## DISCUSSION

PRCA is a rare hematologic condition that may occur for unknown reasons (idiopathic, primary autoimmune disorder) or in association with other immune diseases such as systemic lupus erythematosus, type-1 diabetes *mellitus*, viral infection (parvovirus B19, HIV), lymphocytic leukemia, certain drugs (EPO, carbamazepine, rifampicin, rituximab, mycophenolic acid), pregnancy, and renal failure.^11^

Recombinant human EPO (rhEPO) has been used to treat anemia related to CKD since 1986. The drug has since minimized the risks connected to repeated blood transfusions, including iron overload, transmission of viral hepatitis, and HLA sensitization. Although a significant number of patients have been regularly taking rhEPO, only three cases of PRCA associated to therapy with the drug have been reported within the first few years of its introduction.^12^ However, the number of cases described grew gradually until 1998 and reached maximum incidence in 2001, to then decline until 2003.^13^^-^ 15 All cases described within the period of peak incidence were related to EPO administered subcutaneously, and nearly all patients were then given epoetin alfa (Eprex), a fact probably explained by the use of polysorbate 80 (PS-80) instead of human albumin to stabilize the drug.^16^

In the years of 2001-2003, rates of 46.1/100,000 patients-year for cases of PRCA related to the use of rhEPO were recorded, particularly in connection with Eprex using PS-80 as a stabilizer conditioned in pre-filled syringes with uncoated rubber plungers. These rates were considerably higher when compared to other formulations available at the time.^16^ Similar findings were confirmed in another study published by the US Food and Drug Administration (FDA) in 2004 comparing different formulations of epoetin alfa and epoetin beta (NeoRecormon).^8^ The accumulating evidence led to a temporary suspension of Eprex subcutaneous injections in the years of 2004-2006; the product was later reintroduced with a coated rubber plunger and the likely increase in immunogenicity was attributed to PS-80, since the molecular structure of the various studied epoetins and patient characteristics were not significantly correlated with the events.^17^ The mechanisms involved in the formation of anti-EPO antibodies have been extensively studied. PS-80 and other adjuvant compounds derived from plastics and uncoated plunger materials have been postulated as the most relevant triggers of the process, although protein denaturation and aggregation of other contaminants from low-stability formulations containing PS-80^18^ and failure to keep the drug refrigerated may not be ruled out.

The incidence of PRCA varies depending on the drug, route of administration, and even country of origin. Most of the cases described (92%) by 2004 were connected to subcutaneous EPO and to the most commonly used drug, epoetin alfa. However, the literature also comprises cases involving associations between PRCA and epoetin beta, zetaepoetina,^20^ and darbepoetin alfa,^21^ although in Brazil only epoetin alfa is regularly used.

The diagnostic criteria described for rhEPO-induced PRCA include prior treatment with rhEPO for several weeks; weekly drops in hemoglobin of 1g/dl; need to transfuse one unit of packed red blood cells per week; reticulocyte count below 1%; normal platelet and leukocyte counts in peripheral blood; bone marrow test showing erythroblasts < 5%; presence of circulating neutralizing anti-EPO antibodies; and normal or elevated levels of ferritin and transferrin saturation.^22^

Some initial measures must be taken as part of the therapy for PRCA related to rhEPO, such as offering blood transfusions to address symptomatic anemia and discontinue the medication. Since PRCA is an autoimmune disease, spontaneous remission usually is not a factor despite reports in the literature.^23^ Therefore, patients are usually required to take immunosuppressants. A retrospective study performed with 47 patients who developed neutralizing anti-EPO antibodies reported that none of the nine patients not given specific treatment with immunosuppressants recovered from PRCA. In the group given immunosuppressants, 78% (29 individuals) recovered from the disease after they were offered various protocols including corticosteroids, corticoids and cyclophosphamide or cyclosporine.^24^

Our study described the cases of two patients who developed PRCA while they were being treated with a biosimilar to epoetin alfa, Manguinhos (Fiocruz), the medication then offered by the Ministry of Health. Although both patients responded satisfactorily at the start therapy with EPO, they later progressed with profound anemia and required repeated transfusions and high-dose EPO therapy. In both cases, neutralizing anti-EPO antibodies were found in serum samples.

The patient described in the first case had side effects linked to immunosuppressants. Immunosuppressant therapy was discontinued on account of her advanced age. The patient has been off the target defined for hemoglobin, but has been symptom-free and has not required additional transfusions. The patient on the second case was given similar immunosuppressant therapy - prednisone and cyclosporine - and later underwent renal transplantation at a time when he was in remission from PRCA. He has not relapsed since transplantation. It is important to realize that given the inexistence of specific laboratory support in Brazil to identify anti-EPO antibodies, the blood samples taken from the patients had to be sent to Spain for testing. Therefore, although the patient described in Case 1 may not be categorized as in remission, further antibody titration was not performed on account of the elevated costs connected with performing tests periodically.

In the absence of other causes, it is recommended that patients with CKD on rhEPO with refractory anemia be tested for the presence of anti-EPO antibodies in serum. EPO must be immediately discontinued for patients tested positive, and other types of EPO cannot be offered. Immunosuppressants (combinations of corticosteroids and cyclosporine) may be prescribed as long as there are no contraindications.

Although it is a rare clinical event in patients with CKD treated with rhEPO, PRCA must be remembered as a possible cause of anemia refractory to high-dose EPO therapy after more frequent causes of anemia in populations with CKD have been ruled out. The treatment of cases of PRCA may be challenging, since specific management and laboratory tests are not always readily available.
